# High-temperature epitaxial growth of tantalum nitride thin films on MgO: structural evolution and potential for SQUID applications

**DOI:** 10.3762/bjnano.16.53

**Published:** 2025-05-22

**Authors:** Michelle Cedillo Rosillo, Oscar Contreras López, Jesús Antonio Díaz, Agustín Conde Gallardo, Harvi A Castillo Cuero

**Affiliations:** 1 Centro de Nanociencias y Nanotecnología, Universidad Nacional Autónoma de México (UNAM) km. 107 Carretera Tijuana-Ensenada, Ensenada, Baja California 22800, México; 2 Departamento de Física, Centro de Investigación y de Estudios Avanzados del Instituto Politécnico Nacional (CINVESTAV-IPN), Apdo. Postal 14-740, México D.F. 07360, México

**Keywords:** epitaxial growth, SQUID, structural evolution, superconductivity, TaN thin films

## Abstract

The growth of superconducting tantalum nitride (TaN) thin films on magnesium oxide (MgO) substrates has been studied using pulsed laser deposition (PLD). This research investigates the influence of varying deposition parameters, including substrate temperature and ambient gas composition, on the structural, morphological, and superconducting properties of the films. X-ray photoelectron spectroscopy, X-ray diffraction, atomic force microscopy, and transmission electron microscopy analyses revealed that the TaN films exhibit excellent crystallinity and smooth surface morphology, when deposited at optimal temperatures of 750 and 850 °C. The films exhibit superconducting transition temperatures (*T*_c_) ranging from 5.0 to 6.3 K, depending on the stoichiometry and deposition conditions. Resistance–temperature curves further confirm the high quality of the films, as evidenced by their low residual resistivity ratios. These findings demonstrate that PLD is a suitable technique for producing high-quality TaN superconducting films.

## Introduction

Superconductivity is a quantum mechanical phenomenon characterized by the complete absence of electrical resistance in certain materials when cooled below a critical superconducting transition temperature (*T*_c_). Discovered in 1911 by Heike Kamerlingh Onnes [1], superconductors exhibit unique properties, including perfect conductivity and the Meissner effect [2], that is, the expulsion of magnetic fields. These remarkable properties make them highly valuable for diverse applications, such as lossless power transmission, magnetic levitation in transportation systems, and the creation of powerful electromagnets. Furthermore, superconductivity plays a pivotal role in the development of advanced technologies, including quantum computing and nanometric electronic devices. Despite significant progress, a major challenge remains in discovering materials that exhibit superconductivity at higher, more practical temperatures.

The efficient fabrication of materials with a low superconducting energy gap and an intermediate *T*_c_ is crucial for the development and enhancement of superconducting electronic devices operating in the gigahertz range. Transition-metal compounds, such as nitrides and carbides (e.g., NbN, TiN, TiC, and TaN), have demonstrated *T*_c_ values ranging from 2 to 10.4 K [3–6]. These compounds constitute a significant class of materials because of their exceptional physical and chemical properties, including ultrahardness (comparable to that of diamond) and high melting points around 3000 °C. These properties can be qualitatively understood by observing that the Fermi energy falls within a pronounced minimum of the density of states [7].

Some reports have shown that TaN has a superconductive energy gap lower than that of NbN [7], the most commonly used material for single-photon detectors in the gigahertz range; hence, this material can be a better candidate for superconductive electronic devices. Depending on the amount of incorporated nitrogen, *x*, the tantalum nitride system TaN*_x_* can be an insulator, semiconductor, or superconductor and also can exhibit a variety of crystallographic phases [8–9]. For example, Nie and collaborators mentioned that Ta_2_N thin films presented a high-temperature coefficient of resistance, and resistors using this material as a diffusion barrier with potential application for microelectronics were fabricated [10–11]. In contrast, the stoichiometric mononitride TaN phase with face-centered cubic (FCC) structure exhibits superconductivity with a *T*_c_ of 8.15 K [12]; when the thin films were grown epitaxially on an FCC substrate, *T*_c_ could be pushed up to 10.8 K [13]. The *T*_c_ of TaN depends strongly on the crystallinity and stoichiometry of the thin films. Reports mentioned that pulsed laser deposition (PLD) in the reactive pulsed laser deposition (RPLD) mode is an efficient method for the growth of high-quality thin films [14].

In the present work, superconducting TaN thin films were synthesized using PLD. A high-purity Ta target was ablated in a N_2_ atmosphere while the N_2_ pressure was varied to investigate its effect on film properties. The substrate temperature was systematically altered to explore its impact on the growth dynamics and superconducting characteristics of the films. X-ray diffraction (XRD) analysis revealed that the TaN thin films exhibited excellent crystallinity, with sharp diffraction peaks indicating well-defined structural phases. The deposition process was optimized by systematically adjusting substrate temperature and N_2_ pressure. This optimization aimed to achieve a low oxygen concentration in the films, surpassing levels reported by other researchers. Low oxygen content is crucial for exhibiting superconducting properties and potentially enhancing *T*_c_.

Among the fabricated samples, the film deposited at the optimal combination of above parameters demonstrated the highest *T*_c_ of 6.3 K, signifying superior superconducting behavior. Analysis by XPS manifests the presence of stable oxygen impurities. The oxygen source is the residual gas inside the growth chamber. Quintanar-Zamora et al. provided evidence that oxygen can occupy the N sites of the crystal without structural modifications [15].

These findings reinforce the potential of PLD as a viable method for fabricating high-quality TaN superconducting films with controlled stoichiometry and phase purity. The optimal combination of N_2_ pressure and substrate temperature appears crucial for achieving desirable superconducting performance, making these films promising candidates for applications in superconducting electronics.

## Experimental

TaN thin films were synthesized while systematically varying substrate temperature within the 700–850 °C range and nitrogen pressure between 10 and 90 mTorr. The experiment was performed in a laser ablation system “RIBER LDM 32”. It consists of three stainless steel ultrahigh-vacuum (UHV) chambers for sample introduction, PLD deposition, and X-ray photoelectron spectroscopy (XPS) analysis, isolated by UHV gate valves and independently pumped by ion and Ti sublimation pumps. Every chamber has a base pressure of about 10^−9^ Torr. A Nd:YAG laser with a fourth-harmonic generator (266 nm), was focused onto a 99.999% Ta target (purchased from Kurt J. Lesker Company) with 4 ns and *hv* = 1.5 eV. The laser was operated at a frequency of 7.5 Hz, the substrate-to-target distance was 5 cm, and the incident energy density was 4.88 J·cm^−2^. We studied the stoichiometry and properties of the thin films as a function of gas pressure by introducing N_2_ to realize RPLD. At this time, the ion pumps were closed and isolated from the PLD deposition chamber, and a turbo pump was used to pump the gas out during film deposition. The films were transferred to the analysis chamber to characterize them in situ by XPS. Varying the N_2_ pressure, *p*_N2_, in the chamber and analyzing the films, we obtained TaN thin films at 90 mTorr of N_2_. XPS data were collected using an Al Kα monochromatic source and a hemispherical analyzer from SPECS. The films were grown on (100)-oriented single crystal substrates of magnesium oxide (MgO) (99.9%, purchased from Sigma Aldrich). To obtain a FCC structure, we varied the deposition temperature while using 90 mTorr of N_2_. Prior research has consistently shown that TaN films with a FCC crystal structure exhibit enhanced superconducting properties compared to those with other crystal structures. The XRD spectra were obtained with a Panalitycal X’Pert Pro MRD, and the “Inorganic Crystal Structure Database” powder diffraction database was used for the qualitative search–match phase identification. To determine the epitaxy of the films, TEM was carried out with a Jeol JEM 2100F. Finally, the resistivity, *R*, was measured as a function of the temperature *T* using the van der Pauw method in a DynaCool Quantum Design physical property measurement system. To achieve temperatures below 4.2 K, a vacuum pump was used to reduce the pressure on the helium bath. This technique allows the liquid helium temperature to be lowered to approximately 1.8 K. Reducing the vapor pressure provides an efficient method to reach 2.0 K. Atomic force microscopy (AFM, XE-70 Park Systems) in contact mode was used to study the surface morphology of the films.

The synthesis protocol used in this study was modified from the work reported by Quintanar-Zamora et al. [15] by varying the substrate temperature and the nitrogen pressure.

## Results and Discussion

The atomic concentration of each element (Ta, N, O, and C) in the TaN thin films was determined as a function of *p*_N2_ using XPS (Table 1). Atomic sensitivity factors of 2.5, 0.42, 0.66, and 0.25 were used for Ta 4f, N 1s, O 1s, and C 1s, respectively, during data analysis. XPS measurements focused on the core levels of Ta 4f, N 1s, C 1s, and O 1s, with the peak areas calculated after linear background subtraction. This analysis provided insights into the stoichiometry and impurity levels in the films, which are crucial for optimizing their superconducting and structural properties.

**Table 1:** Atomic composition of TaN thin films as function of *p*_N2_.

	10 mTorr	30 mTorr	50 mTorr	60 mTorr	70 mTorr	90 mTorr

atom % Ta	31.4	24.7	28.1	34.4	30.0	51.1
atom % N	2.4	1.8	5.6	26.6	32.2	36.5
atom % O	62.4	66.3	62.8	32.0	33.8	12.4
atom % C	3.8	6.8	3.5	7.0	4.0	0

The results indicate that nitrogen concentration increases significantly with rising nitrogen pressure. At *p*_N2_ = 70 mTorr, the nitrogen concentration was measured at 32.2 atom %, but this value rose to 36.5 atom % at *p*_N2_ = 90 mTorr. Simultaneously, at 90 mTorr, the oxygen concentration dropped to 10.2 atom %, and the carbon concentration fell to 0 atom %. These reductions in oxygen and carbon impurities are essential for improving the film’s quality, as both elements can introduce defects that degrade the superconducting performance.

At a lower nitrogen pressure of 60 mTorr, the atomic concentrations of Ta and N were found to be nearly equal. However, the concentrations of oxygen and carbon were relatively high at this pressure, which compromises the quality of the TaN films. Consequently, 60 mTorr was discarded as a suitable pressure for synthesizing high-quality films because of the impurity levels.

Although the results for samples grown at 70 mTorr *p*_N2_ show a near-stoichiometric TaN composition, the presence of significant oxygen (32%) and carbon (4%) impurities suggests a composition closer to TaNO. The optimal stoichiometry for TaN thin films was achieved at a *p*_N2_ = 90 mTorr, where the nitrogen concentration reached its highest value, and oxygen and carbon impurities were minimized. These results also align with the results from XRD and TEM, as the film deposited under these conditions exhibited the best crystallinity and epitaxial growth. The combination of optimal stoichiometry and high crystallinity, achieved by controlling both the deposition temperature and nitrogen pressure, plays a pivotal role in enhancing the superconducting properties of the TaN films.

The presence of oxygen in the thin films can be attributed to several factors. Despite rigorous efforts to achieve high-vacuum conditions, a small residual oxygen concentration inevitably remains within the deposition chamber. At elevated temperatures, the reactivity of these residual oxygen molecules increases, facilitating their adsorption onto the growing film surface. However, an increase in N_2_ pressure can lead to the displacement of O_2_, effectively reducing the incorporation of O₂ within the thin films.

The interaction between a laser-generated plume and the surrounding background gas is a dynamic and complex process significantly influenced by the background gas pressure. Understanding these interactions is crucial for optimizing PLD techniques and achieving the desired film properties. At low background pressures, the mean free path of plume particles is substantial, allowing them to travel considerable distances before encountering collisions with background gas molecules. This results in limited interactions and a relatively unperturbed plume expansion. As the background pressure increases, the collision frequency between plume particles and background gas molecules rises. This leads to more pronounced interactions, including increased scattering, energy transfer, and chemical reactions. In the specific case of TaN deposition, increasing the N_2_ background pressure favors the formation of TaN by enhancing the interaction between tantalum species in the plume and the nitrogen gas.

**Figure 1:**
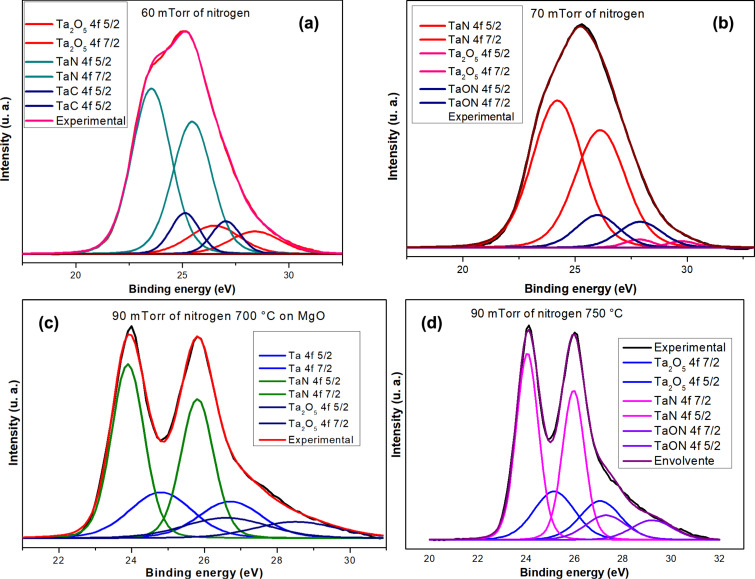
Comparison of Ta 4f XPS peaks determined for different samples. (a) *p*_N2_ = 60 mTorr, (b) *p*_N2_ = 70 mTorr, (c) *p*_N2_ = 90 mTorr, *T* = 700 °C, and (d) *p*_N2_ = 90 mTorr, *T* = 750 °C.

Monitoring the positions of the Ta 4f_7/2_ and Ta 4f_5/2_ peaks can reveal any chemical state changes that Ta and N may undergo during the ablation process. Figure 1 presents the deconvolution of high-resolution XPS peaks for Ta 4f, shown both before (Figure 1a,b) and after (Figure 1c,d) heating the substrate. The peaks were fitted using Gaussian functions. The relative atomic concentrations of Ta and N were calculated from the areas under the Ta 4f and N 1s peaks after Touggard background subtraction, using CasaXPS software.

As shown in Figure 1, at *p*_N2_ = 60 mTorr, we see contributions from tantalum oxide, tantalum nitride, tantalum carbide, and metallic tantalum. At 70 mTorr, contributions from these compounds are still present (33.8 atom % O), along with tantalum oxynitride. Shi et al. [16] synthesized TaN nanocrystals and reported that the TaN 4f_7/2_ XPS peak appears at 23.5 eV. Similarly, Bayer and collaborators [17] identified the TaC 4f_7/2_ XPS peak at 23.3 eV, and Yang et al. [18] reported a photoemission peak at 25 eV.

We found that increasing the nitrogen flow substantially reduces the amount of oxygen in the films (Table 1) compared to that reported by Quintanar-Zamora et al. [15]. The oxygen content remains almost invariable when increasing the temperature to 850 °C (Table 2). Although higher temperatures can promote the desorption of impurities, including oxygen, in this case, the temperature range between 700 and 850 °C is not sufficient to drastically change the rate of oxygen incorporation or removal. Thus, any further increase in temperature beyond 700 °C does not affect oxygen content due to limited oxygen availability and the dominant role of nitrogen flow.

**Table 2:** Effect of substrate temperature on the atomic composition of TaN thin films (*p*_N2_ = 90 mTorr).

Deposition temperature	atom % Ta	atom % N	atom % O

700 °C	51.0	36.6	12.5
750 °C	51.9	34.8	13.3
800 °C	48.2	38.8	13.0
850 °C	47.6	37.1	15.3

The samples in Figure 1c,d present a stoichiometry closer to that of TaN than the smaples in Figure 1a,b; they have an atomic composition of Ta_2_O_5_, and the doublet shape is more defined. Table 2 shows the atomic composition of samples heated at 700 to 850 °C using *p*_N2_ = 90 mTorr. The tantalum concentration does not vary much as the temperature changes, but the concentrations of nitrogen and oxygen do vary. The incorporation of oxygen into TaN was studied in detail by Quintanar-Zamora and collaborators [15].

**Figure 2:**
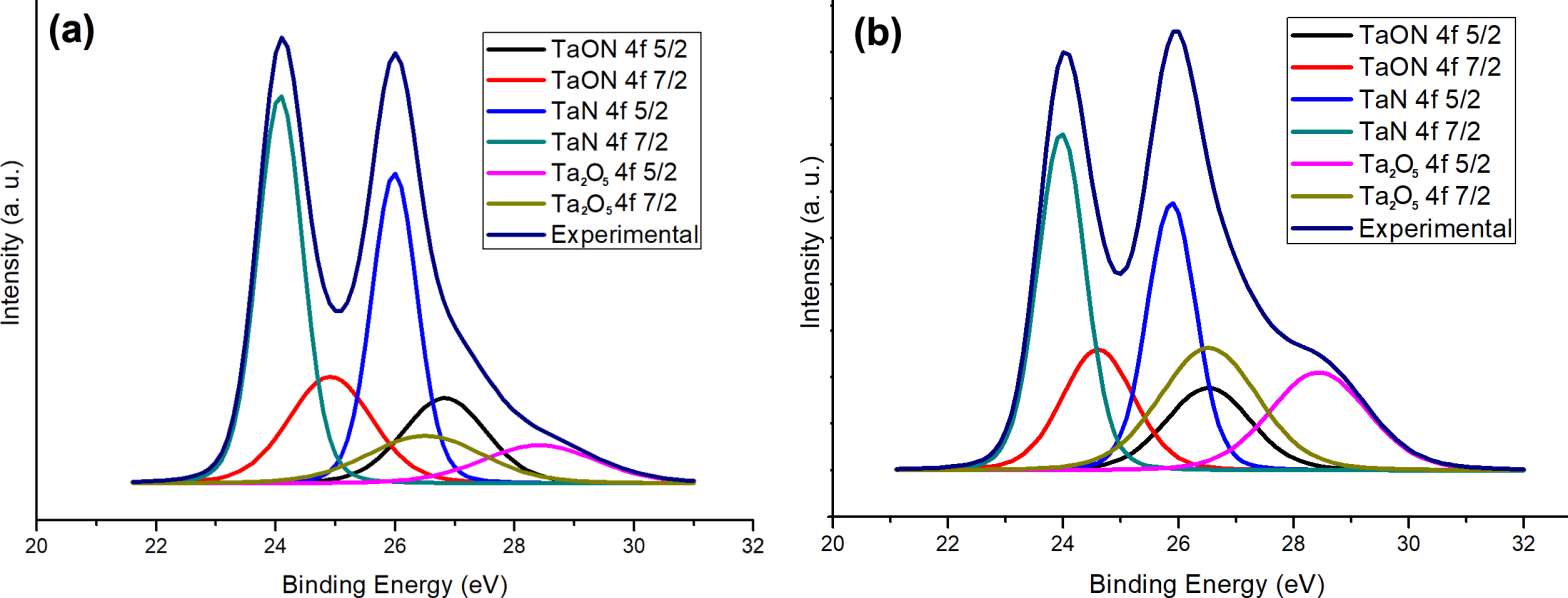
Ta 4f XPS spectra of samples deposited at (a) *p*_N2_ = 60 mTorr, *T* = 800 °C and (b) *p*_N2_ = 60 mTorr, *T* = 850 °C.

When the temperature is increased, tantalum allows for a greater incorporation of oxygen, forming lattice interstitials. The increased incorporation of oxygen into tantalum at higher temperatures is a complex phenomenon driven by a combination of factors, including increased atomic mobility, enhanced thermodynamic driving forces, and the availability of interstitial sites within the lattice. It is confirmed by the concentration of the sample heated to 850 °C (Table 2) and by the Ta_2_O_5_ doublet’s intensity in Figure 2b; the intensity of the Ta_2_O_5_ doublet in Figure 2a is lower than that in Figure 2b.

**Figure 3:**
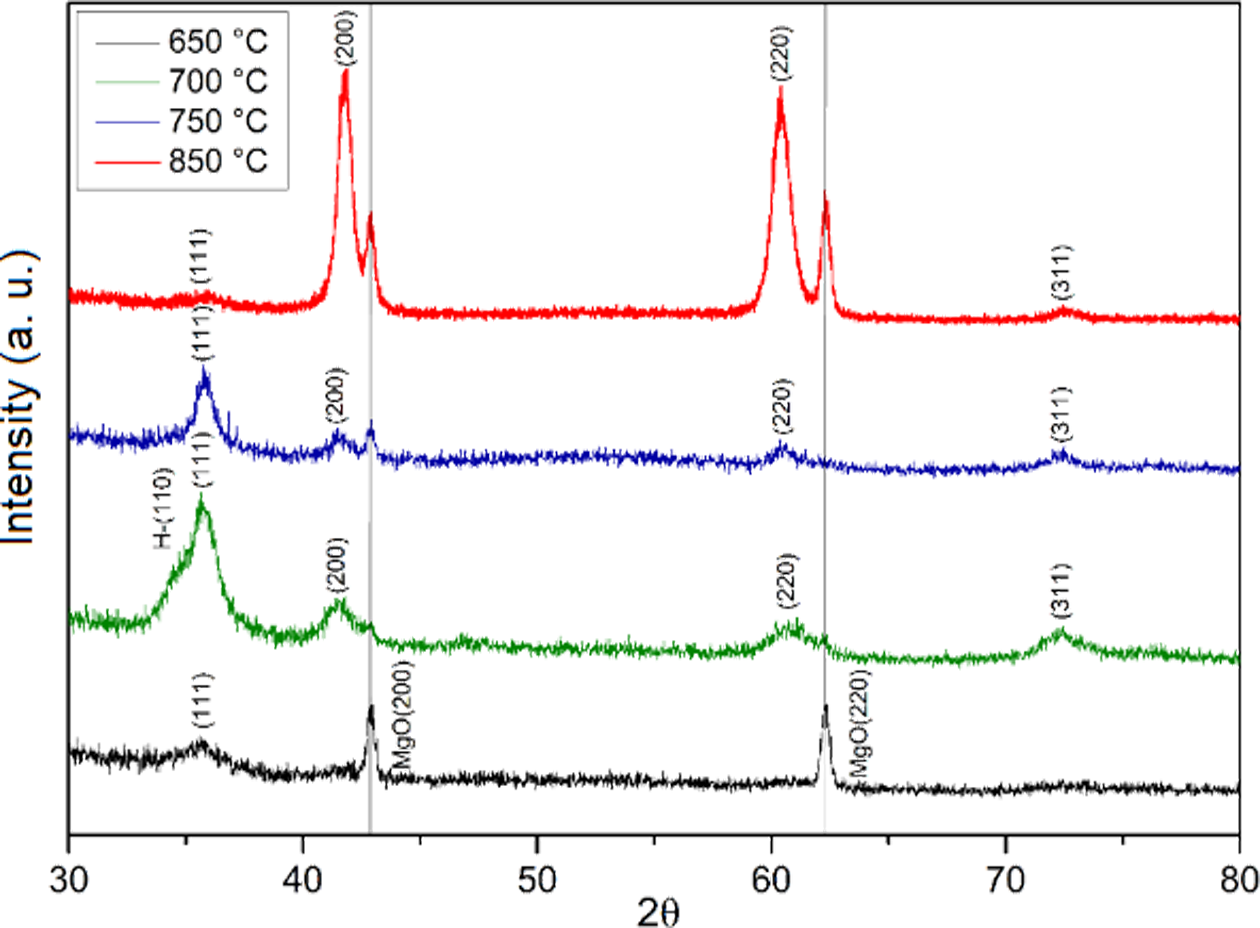
XRD spectra of TaN thin films on MgO substrates for deposition temperatures of 650, 700, 750, and 850 °C and *p*_N2_ = 90 mTorr.

The effect of the deposition temperature on the structural evolution of TaN thin films deposited on MgO(100) substrates was investigated using XRD and TEM. Figure 3 shows the XRD patterns of TaN films deposited at various temperatures, revealing critical insights into phase development, crystallinity, and epitaxial growth as the temperature increased.

For a deposition temperature of 650 °C, the XRD results show a low-crystallinity film, as evidenced by the weak intensity of the δ-TaN(111) reflection. The FCC structure of the δ-TaN phase is identified by its characteristic (111), (200), (220), and (311) reflections, based on JCPDS card no. 49-1283. Additionally, a weak (110) reflection of the β-TaN phase (JCPDS card no. 039-1485) is present, though the overall crystallinity is poor at this deposition temperature. As the deposition temperature is increased to 700 °C, a significant change in the film’s phase composition is observed. A mixed phase of δ-TaN and β-TaN appears, with the cubic δ-TaN phase becoming more prominent compared to the hexagonal β-TaN phase. The increased crystallinity is evident from the sharper and more intense diffraction peaks. The coexistence of both cubic and hexagonal phases at 700 °C suggests an intermediate stage of structural evolution, where the cubic δ-TaN phase begins to dominate the overall microstructure.

At 750 °C, the XRD patterns show a further increase in the dominance of the cubic δ-TaN phase, indicating a structural transition towards a more stable phase at higher temperatures. The β-TaN phase is significantly reduced, and the δ-TaN phase becomes the major phase present in the film. This increased phase purity and the sharper diffraction peaks indicate enhanced crystallinity, aligning with the structural requirements for high-performance superconducting films.

The most significant structural improvement was observed at a deposition temperature of 850 °C. At this temperature, the XRD pattern confirms the epitaxial growth of the TaN thin film on the MgO substrate. This epitaxial growth is facilitated by the similar crystallographic structures of TaN and MgO (both cubic) and their relatively small lattice mismatch. The lattice parameter of TaN is approximately 4.33 Å, while that of MgO is 4.21 Å, resulting in a lattice mismatch of 2.85%. This small mismatch suggests that epitaxial growth is possible, with a moderate level of strain expected within the TaN film.

The (200) and (220) reflections of the δ-TaN phase show high intensity, while the (111) and (311) reflections exhibit lower intensity. The sharpness and intensity of these peaks indicate a highly crystalline film with excellent alignment to the MgO (100) substrate. The epitaxial relationship between film and substrate is confirmed by the matching orientations of the lattice planes, suggesting a coherent interface between the two materials. Similar results were reported by Chaudhuri et al. [19] and Elangoval and collaborators [20].

The XRD patterns reveal a relatively low contribution from the Ta_2_O_5_ and TaNO compounds, which is corroborated by the XPS spectra. This observation can be attributed to the structural similarity between these compounds and TaN. Specifically, the reflections of the (111) planes of FCC Ta_2_O_5_ and TaNO exhibit significant proximity to the reflection of the same planes in TaN, also with an FCC structure, as reported by Hagiwara and collaborators [21]. This overlap of reflections complicates the precise deconvolution of the minor phases in the XRD diffractograms, underscoring the need to carefully consider potential peak coincidences when analyzing multiphase systems containing these tantalum compounds.

**Figure 4:**
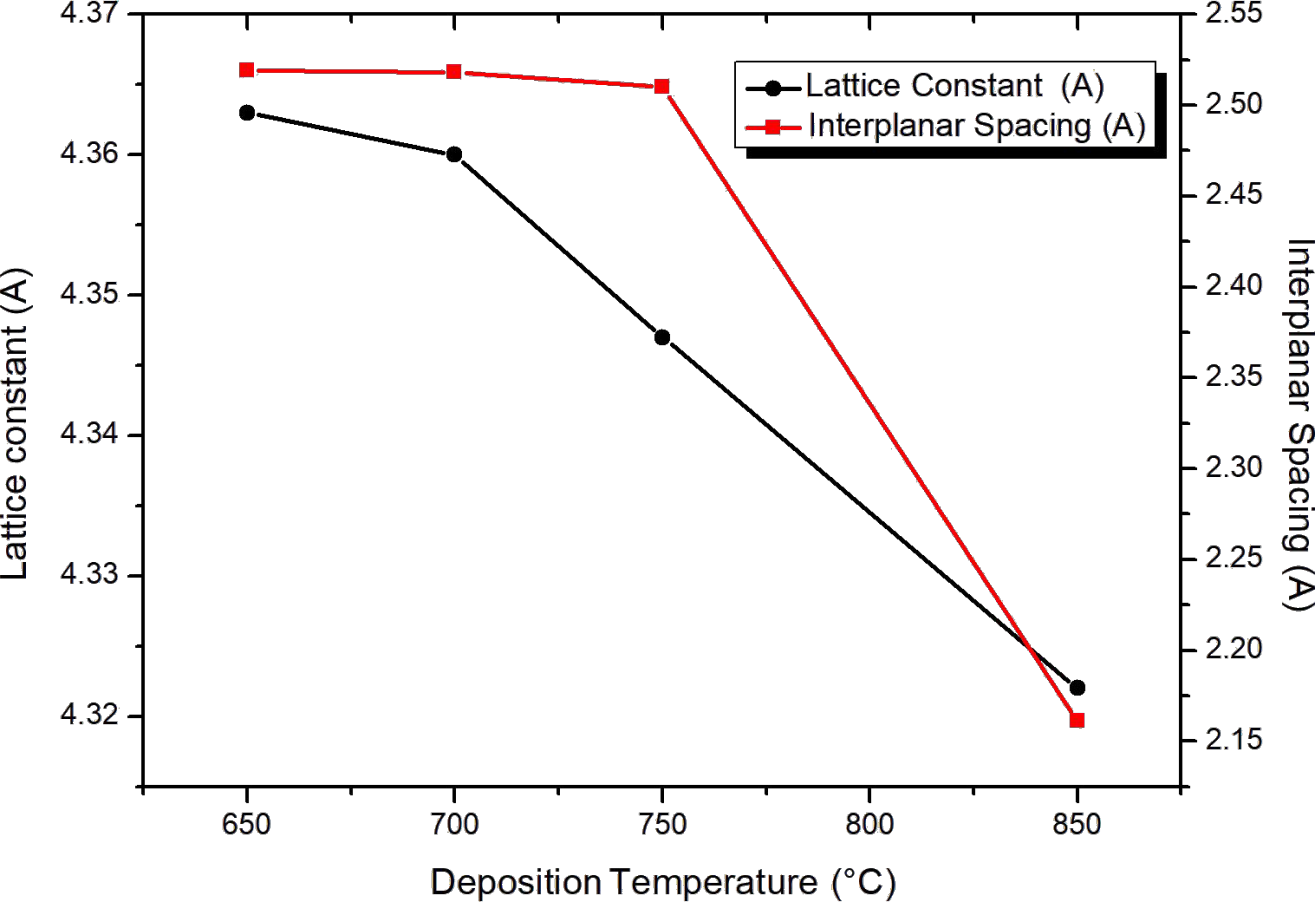
Lattice constant and interplanar spacing of TaN thin films deposited at different temperatures of 650, 700, 750, and 850 °C and *p*_N2_ = 90 mTorr.

Figure 4 shows how the lattice parameter and interplanar spacing decreased as the temperature deposition increased. The lattice parameter decreased from 4.36 to 4.32 Å, as the temperature deposition increased. This is attributed to increased atomic mobility and improved crystallinity during growth. In fact, at 850 °C, the adatom surface mobility and surface diffusion of the deposited atoms are further increased, which has also been shown by Elangovan et al. [20], Adamik et al. [22], and Cheng and collaborators [23].

**Figure 5:**
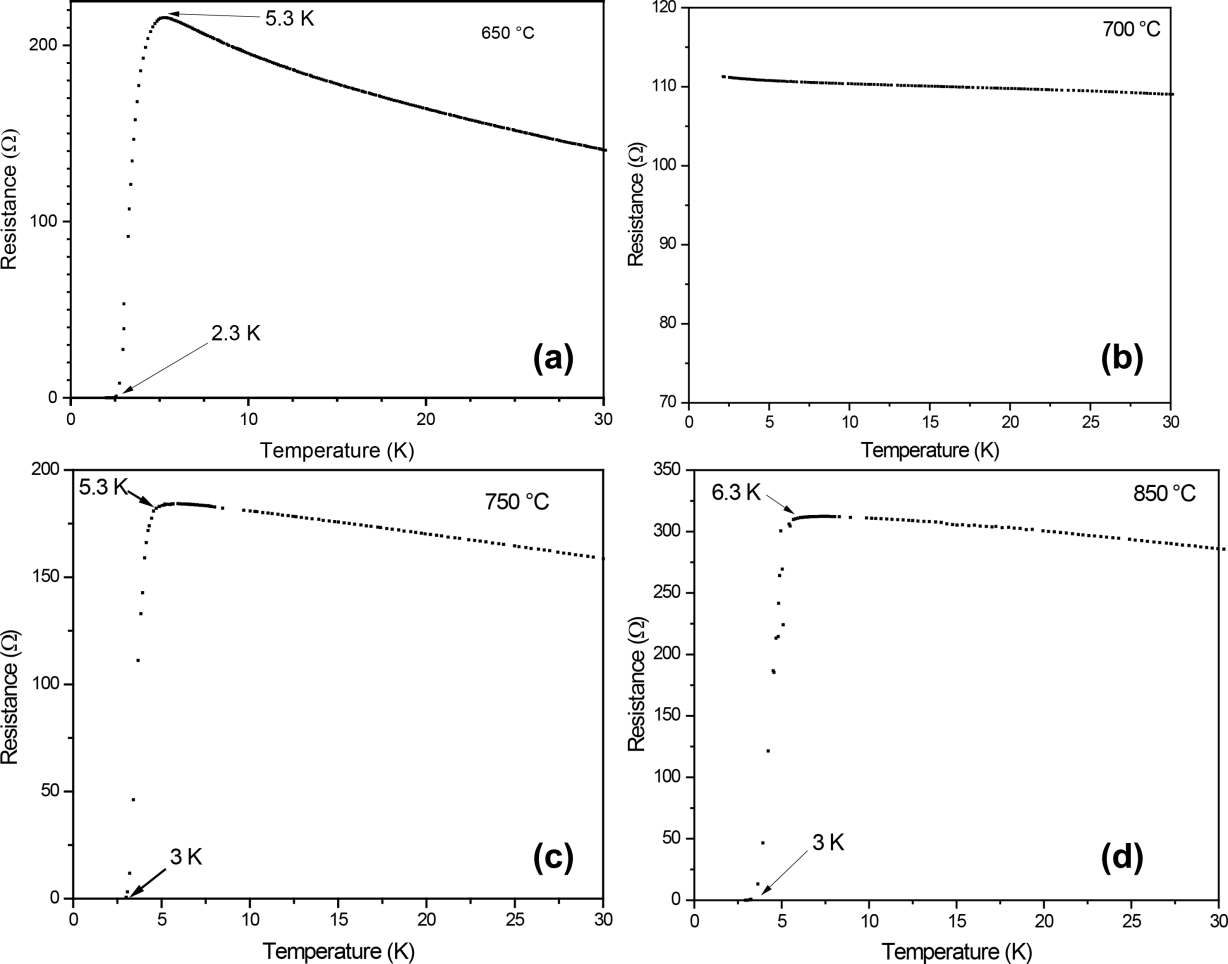
Resistance of TaN films grown at *p*_N2_ = 90 mTorr as a function of the deposition temperature: (a) 650 °C, (b) 700 °C, (c) 750 °C, and (d) 850 °C.

The variation of resistance of TaN films, measured from room temperature down to liquid helium temperature, and the *T*_c_ values are shown in Figure 5. As mentioned previously, a mixture of δ-TaN and β-TaN was identified in the film deposited at 700 °C. The mixture of these phases results in the absence of superconductivity [24]. Upon increasing the deposition temperature to 750 °C, the sample, which has δ-TaN as the significant phase, exhibits a superconducting transition at *T*_c_ = 5.3 K. The highest value of *T*_c_ was obtained for the film deposited at 850 °C with *p*_N2_ = 90 mTorr (*T*_c_ = 6.3 K).

**Figure 6:**
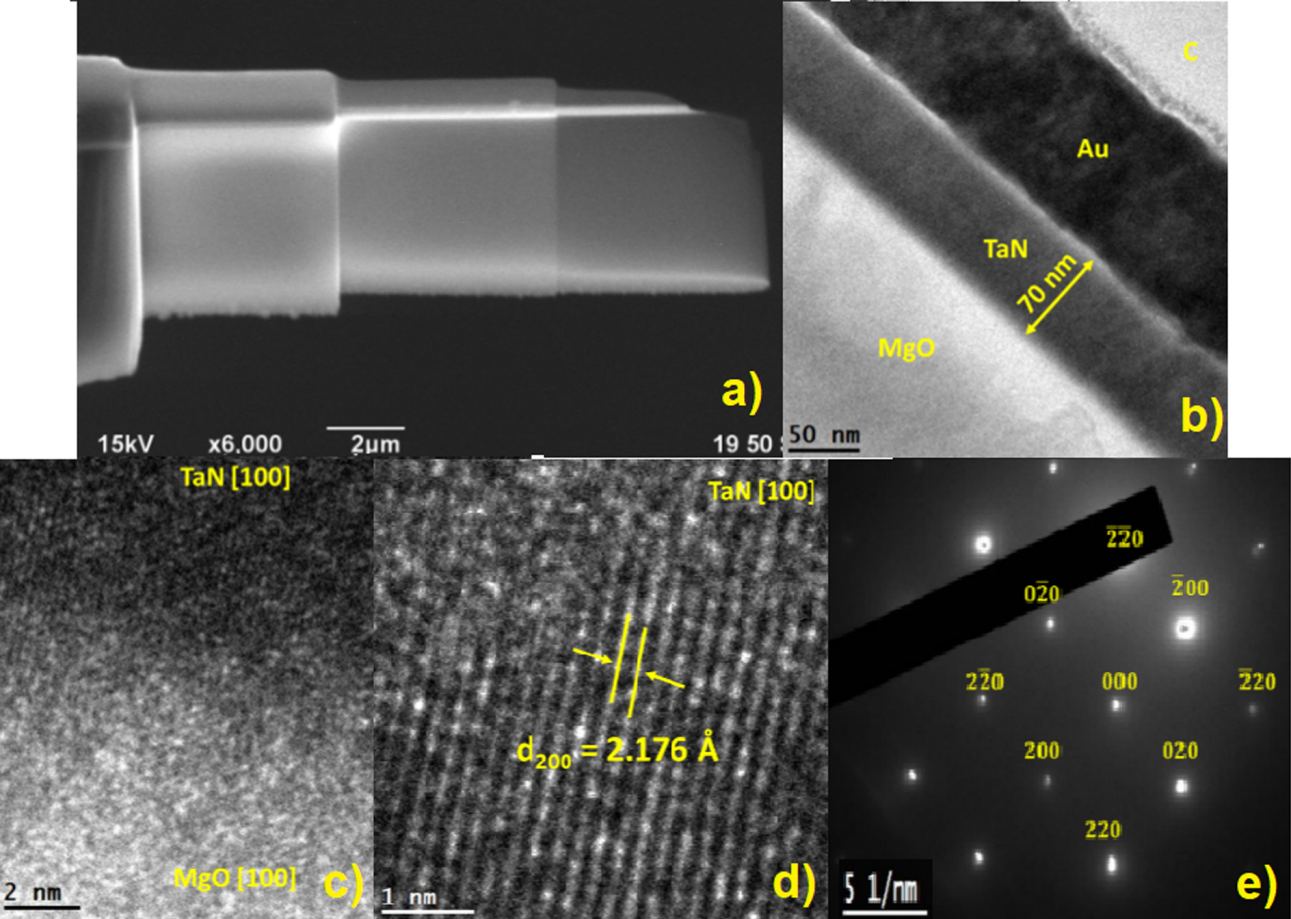
(a) SEM image of the cross section of a TaN thin film prepared with FIB. (b) TEM analysis of the lateral region of the TaN film deposited at *T* = 750 °C and *p*_N2_ = 90 mTorr. (c) TEM image at the interface between MgO substrate and TaN thin film. (d) Transversal section showing the interplanar spacing of TaN. (e) Indexed electron diffraction pattern of the TaN thin film.

Cross-sectional transmission electron microscopy samples were prepared by a 4 kV Ar focused ion beam (FIB) for SEM observation; Au films were evaporated on the TaN samples before the FIB process to protect the samples (Figure 6a). The sample in Figure 6b shows a film thickness of 70 nm, and one can distinguish the Au protective layer used for the FIB process. Figure 6c shows the epitaxial growth in the MgO [100] direction, and Figure 6d shows a region away from the substrate. There are low-crystallinity regions and others that have planes with a growth in the direction of the substrate. Figure 6e shows the electron diffraction pattern of TaN; it presents the (200) and (220) planes because the sample contains TaN as a major phase.

TEM analysis provides further confirmation of the epitaxial growth and crystallinity of the δ-TaN thin films. The interplanar spacing was calculated using Digital Micrograph software for both the MgO substrate and the TaN film. For the substrate, the interplanar spacing was found to be *d* = 2.115 Å, while the film’s interplanar spacing was slightly larger, *d* = 2.176 Å. This slight mismatch is typical for epitaxial growth, where the film adapts to the lattice parameters of the substrate (Figure 6c).

In regions farther from the substrate, the interplanar spacing was measured as *d* = 2.169 Å, indicating that the FCC structure persists throughout the film, maintaining a high degree of crystallinity. Figure 6d and Figure 6e confirm the presence of the FCC phase with clear identification of the (200) and (220) planes, consistent with the XRD results. The film’s calculated interplanar spacings closely match the values obtained from the XRD analysis, further validating the structural data.

Overall, the films deposited at 750 and 850 °C exhibit the highest degree of crystallinity, as demonstrated by both XRD and TEM analysis. The epitaxial alignment of the TaN film with the MgO substrate is critical for achieving superior superconducting properties, as high crystallinity and minimal defects reduce electron scattering, improving *T*_c_ of the film. The combination of cubic phase dominance, epitaxial growth, and precise lattice match with the MgO substrate makes this film an excellent candidate for superconducting applications, particularly in devices such as superconducting quantum interference devices (SQUIDs).

**Figure 7:**
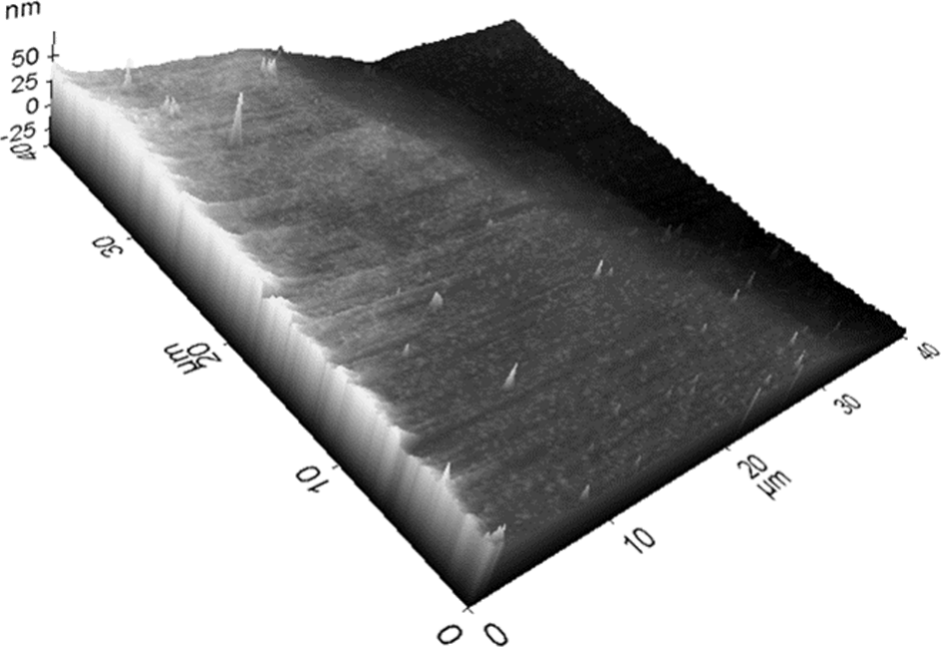
AFM image of the TaN film deposited at *p*_N2_ = 90 mTorr and *T* = 850 °C.

Figure 7 presents an AFM image revealing the low surface roughness (2.2 nm) of even the film deposited at an elevated growth temperature of 850 °C. This observation suggests that while deposition parameters significantly influence the structural and superconducting properties of the TaN thin films, they had only minimal impact on the surface roughness. Notably, the film roughness remains largely unaffected by variations in growth temperature.

## Conclusion

Tantalum nitride thin films were synthesized via reactive pulsed laser deposition. The films exhibit different superconducting transition temperatures depending on deposition temperature and stoichiometry. Films grown at 650 and 700 °C do not show superconductivity; in contrast, films grown at 750 and 850 °C have critical temperatures of 5.3 and 6.3 K, respectively. XPS results show the presence of oxygen, which is difficult to eliminate; even so, these films are superconducting. The presence of oxygen does not cause the samples to lose superconductivity, as long as their structure remains cubic. Quantification shows that tantalum allows for a greater incorporation of oxygen at high temperatures.

XPS analysis demonstrated that the nitrogen pressure critically influences both stoichiometry and impurity content of the TaN thin films. A nitrogen pressure of 90 mTorr during deposition provides the best stoichiometry while minimizing the levels of oxygen and carbon impurities. This pressure, in combination with an optimal deposition temperature of 850 °C, results in the growth of high-quality, epitaxial TaN films with excellent structural and superconducting characteristics.

The TEM and XRD analyses suggested that further refinement of the deposition process, such as eliminating oxygen impurities and fine-tuning of the nitrogen stoichiometry, could result in even higher critical temperatures. The epitaxial TaN film synthesized at 850 °C exhibits superconducting properties with a critical temperature of 6.3 K. In materials with superconducting properties, any variation of an external magnetic field induces electric currents on the surface that counteract this variation. This unique property enables the materials to detect very weak magnetic fields. This principle is leveraged in developing SQUIDs, which are highly sensitive instruments designed to measure extremely weak magnetic fields. The operation of a SQUID is intimately linked to the fundamental properties of superconductors and their interaction with magnetic fields. Therefore, the results of this work demonstrate the potential of thin TaN films for integration into advanced superconducting applications.

AFM analysis confirmed the smooth surface morphology of the films, suggesting that the deposition parameters significantly influence both the structural and superconducting properties of the TaN thin films, but not the surface roughness.

## Data Availability

Data generated and analyzed during this study is available from the corresponding author upon reasonable request.
